# Decreased photosynthesis in the *erect panicle 3* (*ep3*) mutant of rice is associated with reduced stomatal conductance and attenuated guard cell development

**DOI:** 10.1093/jxb/eru525

**Published:** 2015-01-11

**Authors:** Hongyang Yu, Erik H. Murchie, Zinnia H. González-Carranza, Kevin A. Pyke, Jeremy A. Roberts

**Affiliations:** Plant and Crop Sciences Division, School of Biosciences, University of Nottingham, Sutton Bonington LE12 5RD, UKUK

**Keywords:** *Arabidopsis*, *ERECT PANICLE3*, F-box protein, guard cell, *HAWAIIAN SKIRT*, photosynthesis, rice, stomatal conductance.

## Abstract

The *ERECT PANICLE 3* gene of rice encodes a peptide that exhibits more than 50% sequence identity with the *Arabidopsis* F-box protein HAWAIIAN SKIRT (HWS). Ectopic expression of the *Os02g15950* coding sequence, driven by the *HWS* (*At3g61950*) promoter, rescued the *hws-1* flower phenotype in *Arabidopsis* confirming that *EP3* is a functional orthologue of *HWS*. In addition to displaying an erect inflorescence phenotype, loss-of-function mutants of *Os02g15950* exhibited a decrease in leaf photosynthetic capacity and stomatal conductance. Analysis of a range of physiological and anatomical features related to leaf photosynthesis revealed no alteration in Rubisco content and no notable changes in mesophyll size or arrangement. However, both *ep3* mutant plants and transgenic lines that have a T-DNA insertion within the *Os02g15950* (*EP3*) gene exhibit smaller stomatal guard cells compared with their wild-type controls. This anatomical characteristic may account for the observed decrease in leaf photosynthesis and provides evidence that *EP3* plays a role in regulating stomatal guard cell development.

## Introduction

Inflorescences of the *erect panicle 3* (*ep3*) mutant in rice remain upright throughout grain filling. The phenotype is associated with an elevation in the number of small vascular bundles and an increase in the thickness of the parenchyma in the peduncle and a reduction in the number of spikelets per panicle. The *EP3* gene has been cloned and the *ep3* mutation attributed to a single base pair change in *Os02g15950* that leads to the introduction of a premature termination codon (Piao *et al.*, 2009).

A potential orthologue of *EP3* in *Arabidopsis* is the gene *HAWAIIAN SKIRT* (*At3g61950*). The protein encoded by *EP3* shares in excess of 50% sequence homology with HWS at the amino acid level. The *hws-1* mutant is distinctive as its floral organs look as if they are attached to the developing silique throughout maturation and senescence. A detailed study of flower development has revealed that this is the consequence of the sepals being fused into a single whorl trapping the separated petals and anther filaments and preventing them from being shed (González-Carranza *et al.*, 2007). Identification and characterization of the gene responsible for the mutant phenotype has revealed a 28bp deletion in a gene encoding an F-box protein leading to the synthesis of a truncated HWS peptide (González-Carranza *et al.*, 2007). Phenotypic studies of *hws-1,* and transgenic lines ectopically expressing *HWS*, have shown that the gene may also regulate both the size of aerial organs and seeds (González-Carranza *et al.*, 2007) and play a role in regulating stomatal distribution and aspects of chloroplast assembly (Z González-Carranza *et al*., unpublished data).

There is, therefore, reason to believe that the *EP3* gene in rice (*Oryza sativa*) may have a role in determining anatomical, stomatal, and photosynthetic properties. There are known relationships in many species between photosynthetic rate and features of leaf anatomy such as vein density and mesophyll cell properties (McKown and Dengler, 2007; Flexas *et al.*, 2012; Feldman *et al.*, 2014). Stomatal properties are especially important in the context of balancing CO_2_ uptake with transpirational water loss and gaseous exchange through stomatal pores is optimized via a series of signalling pathways which have been well studied in *Arabidopsis* and other species (Raven, 2002; Bergmann and Sack, 2007; Kim *et al.*, 2010). Over 60 genes have been reported to regulate stomatal development in *Arabidopsis* and detailed functional analysis has indicated that these contribute to cell-fate specification, cell polarity, cell division, and cell–cell communication (MacAlister *et al.*, 2006; Ohashi-Ito and Bergmann, 2006; Pillitteri *et al.*, 2006; Pillitteri and Torii, 2012). The mechanisms responsible for stomatal development and signal transduction in rice have been less well studied. However, it has been highlighted recently that both stomatal and mesophyll conductance have an effect on photosynthetic potential in this crop species (Kusumi *et al.*, 2012; Adachi *et al.*, 2013).

In this study, our attention was focused on the physiological impact of the *EP3* gene on photosynthesis in rice as there is evidence that its putative orthologue in *Arabidopsis* can influence stomatal development. Our results show that *EP3* is a functional orthologue of *HWS* and that the gene has a role in regulating rice photosynthesis via stomatal development. This study has practical as well as fundamental implications as rice is one of the most important crops in the world and feeds over half of the world’s population. Further improvement of potential yield in rice is considered to require an increase in photosynthetic efficiency (Zhu *et al.*, 2010). However, the improvement of rice photosynthesis and water use efficiency is a major challenge for crop physiologists and an understanding of how leaf morphology, stomatal development, and guard cell aperture is controlled is integral to this goal (Zhang, 2007; Xing and Zhang, 2010; Zhu *et al.*, 2010; Sharma *et al.*, 2013). Detailed gene function studies are necessary to understand further the regulation of rice leaf photosynthesis and the impact on final yield.

## Materials and methods

### Plant materials and growth conditions

Seeds of the rice mutant *erect panicle3* (*ep3*), and a T-DNA insertion line 1C-03432 were kindly supplied by Dr Hee-Jong Koh (Seoul National University) together with seeds of their wild-type controls. The *ep3* mutant was generated by *N*-methyl-*N*-nitrosourea mutagenesis of Hwasunchalbyeo seed resulting in the introduction of a single base-pair change in the second exon (G/C–A/T) leading to the conversion of a codon for tryptophan into a premature stop (Piao *et al.*, 2009). Hwayoungbyeo plants contained a T-DNA insertion, sited between 1921bp and 1922bp downstream of the ATG, that disrupted the putative second exon of *Os02g15950*.

Rice plants were grown in a growth room at 28 °C with a 12/12h light/dark photoperiod and illuminated by a bank of 400W metal halide lamps, a light intensity at plant height of 400 µmol m^–2^ s^–1^ and a relative humidity of approximately 50%. Rice seeds were placed on filter papers moistened with distilled water in Petri dishes to promote germination and were then transferred to 1.5ml Eppendorf tubes with their bottoms removed and floated on a hydroponic nutrient solution (Murchie *et al.*, 2005) until the emergence of the second leaf. They were then transferred to a hydroponic tank system which was used for the further growth of rice plants and the nutrient compositions were as described in Murchie *et al.* (2005). Plants were held in place via sponges through 3cm diameter holes. Light-resistant materials were used to inhibit the growth of algae. Tap water was used to make up the nutrient solution and the final pH was adjusted to between 5.0 and 5.5 using HCl. Fully expanded leaf 6 was used throughout for the measurements.

Plants of *Arabidopsis* including Col-0 and the *hawaiian skirt-1* (*hws-1*) mutant were grown in plastic pots or plastic trays containing Levington M3 compost under growth-room conditions of 20/4h light/dark at 23±1 °C. Intercept at 0.2g l^–1^ was added to the soil to prevent any attack of the plants by insect larvae.

### Genomic PCR

The primers FCAPsEP3 (see Supplementary Fig. S1 at *JXB* online) were modified from Piao *et al.* (2009) to improve amplification efficiency and used to genotype the *ep3* MNU mutant. Primers F2EP3, R2EP3, and FLBC 5′ were designed to genotype the T-DNA insertion line (see Supplementary Fig. S1 at *JXB* online). A Polymerase Chain Reaction (PCR) was performed using MangoTaq™ DNA polymerase (BiolineTM) according to the manufacturer’s instructions and programmes were run using a GeneAmp PCR system 9700 (Applied Biosystems). The PCR parameters used were: 94 °C for 5min; 32 cycles of 94 °C for 15 s, 58 °C for 30 s, and 72 °C for 1min. A final elongation step was performed at 72 °C for 7min.

### Plasmid construction and plant transformation

Rice Hwasunchalbyeo genomic DNA was extracted using the Qiagen DNAeasy Plant Mini kit (Qiagen) following the manufacturer’s instructions and a genomic region of 1227bp containing an open reading frame beginning in the second predicted exon 2 was amplified using PhusionTM High-Fidelity DNA Polymerase (New England BioLabs) and sub-cloned into the vector pBI101.2:*HWS*_*pro*_ (González-Carranza *et al.*, 2007) to perform a complementation test in *Arabidopsis hws-1* mutant plants. The first predicted exon 1 was not included as all other putative *HWS* orthologues have only a single exon. The primers used were: ForEP3 and RevEp3 (see Supplementary Fig. S1 at *JXB* online); a second set of primers was designed to sub-clone this genomic region containing the restriction sites *Bam*HI and *Sma*I in the forward and reverse primers, respectively: ForEP3*Bam*HI and RevEP3*Sma*I (see Supplementary Fig. S1 at *JXB* online) The *E.coli* DH5α competent cells were transformed and positive colonies from kanamycin selective plates were tested by PCR. Plasmids were extracted from the PCR-confirmed colonies using QIAprep® Miniprep Kit (Qiagen) and sequenced with primers ForHWS5UTR and RevGUS90 (Eurofin).

Positive plasmids were electroporated into *Agrobacterium tumefaciens* strain C58 and transformed into *Arabidopsis* plants *hws-1* and Col-0 using the floral dip method (Clough and Bent, 1998). T_1_ plants were screened on MS plates containing kanamycin at a final concentration 50ng ml^–1^ and were grown at 22 °C with 24h daylight. Antibiotic-resistant plants were transplanted into soil under the *Arabidopsis* growth conditions described in the Pplant materials and growth conditions section. Genotyping was carried out using primers SSLPHSfor and SSLPHSrev (see Supplementary Fig. S1 at *JXB* online) to test the *hws-1* background and F2EP3 and RevGUS90 (see Supplementary Fig. S1 at *JXB* online) to test the cloned ORF from the rice *EP3* gene.

### Gas-exchange and chlorophyll measurements

The fully expanded leaf 6 of rice plants was used for gas-exchange measurements using a Li-Cor 6400 XT Portable Photosynthesis System with chlorophyll fluorescence attachment (Li-Cor Biosciences, USA). Measurements were taken in the growth room between 10.00h and 15.00h. The settings were as follows: flow rate 500 µmol s^–1^, block temperature 30 °C with ambient relative humidity. To determine the light-response curve, the sample (cuvette) CO_2_ concentration was set to 400 µmol mol^–1^ and 10 points of lamp PAR were used varying between 0 and 2000 µmol m^–2^ s^–1^ (10% blue). Apparent quantum yield, light compensation points, light-saturated photosynthetic capacity, and dark respiration were measured using the resulting light-response curve.

Assimilation versus internal leaf CO_2_ concentration (*A*/*C*_i_) curves were performed to gain information on the carboxylation capacity (*V*_cmax_), electron transport capacity (*J*_max_), and the proportion of photosynthesis limited by stomatal conductance (*l*). The flow rate was set to 500 µmol s^–1^, the block temperature was set to 30 °C, and lamp PAR was set to 500 µmol m^–2^ s^–1^ (10% blue). A series of reference cuvette CO_2_ concentrations was used.

To explore stomatal conductance (*g*_s_) responses to changes in relative humidity, gas exchange measurements were performed using manually set levels of cuvette humidity. The setting was as above for the light-response curves but with a PAR of 500 µmol m^–2^ s^–1^ (10% blue). The relative humidity (RH) was set to 60%, 55%, 50%, 45%, and 40% by manually adjusting the desiccant bypass/scrub when measurements were taken. Leaves used for measurements were left in the chamber under the respective RHs until the values of photosynthesis and stomatal conductance stabilized, usually about 2–3min. Leaf temperature was set to 25 °C. The PAR was first set to 2000 µmol m^–2^ s^–1^ (10% blue) for about 1min to make stomata open fully, and then set to 500 µmol m^–2^ s^–1^ (10% blue) for taking measurements. Manual change of humidity was performed when the *g*_s_ value stabilized at a PAR of 500 µmol m^–2^ s^–1^.

Leaf chlorophyll content was estimated using a SPAD-502 chlorophyll meter (Konica, Minolta) before the gas-exchange measurement (Markwell *et al.*, 1995). Five points on each leaf were used to calculate a mean SPAD value for each biological replicate.

Dark adapted *F*_v_/*F*_m_ was determined using a Mini-PAM (Heinz Walz GmbH) at the widest region through the leaf (the middle of the leaf). A 1h dark adaption was performed for the plant leaves using a dark leaf clip DLC-8 (Heinz Walz GmbH).

### Leaf anatomical analysis

A silicon based impression resin Coltene President Plus Jet (Colténe Whaledent, Switzerland) was used for leaf surface impression to determine stomatal density and stomatal size (Smillie *et al.*, 2012). The two components of the resin were mixed in a Petri dish just before application on the leaf surface using a plastic stick. The resin impression was removed from the leaf surface after 10min and then painted with a layer of clear nail varnish. The nail varnish was then peeled off from the resin and put onto a glass slide for further imaging under a light microscope (Nikon optiphot-2) with a Leica DFC 320 camera. Stomatal guard cell length and width were measured using the straight line in Image J software while stomatal guard cell area (the area of two stomatal guard cells when stomata were fully shut so that the influence of stomatal aperture was avoided) was measured using the freehand selection area in Image J (Abràmoff *et al.*, 2004). Subsidiary cells were not included in these measurements. All stomata were closed when measurements were taken to avoid an influence of aperture on the measurement.

Anatomical characters were determined from leaf transverse sections. The leaf sample was positioned in a raw potato cube and a razor blade was used for hand cutting. Sections were then mounted on a microscope slide and then cleared using a solution of 85% (w/v) lactic acid in chloral hydrate. Slides were put into Petri dishes in a 70 °C water bath for 1h. Distilled water was used to wash the sections after incubation and a solution containing 1% (w/v) toluidine blue in 1% (w/v) disodium tetraborate was used for staining. Sections on the slide were placed in dye solutions for 15 s before washing several times with distilled water until they were ready to be observed under a microscope (Smillie *et al.*, 2012). A Leica DMRB microscope with a Leica EC3 camera was used to take photographs. The photographs of the leaf sections were analysed and measured using Image J software.

Separated single cell preparation was generated (Pyke and Leech, 1987; Smillie *et al.*, 2012). A 1mm wide piece of the leaf samples was fixed in a 3.5% (w/v) glutaraldehyde solution for 1h in the dark and then transferred to a solution of 0.1M NaEDTA (pH 9.0) before incubating at 60 °C for at least 6h. These samples were then left at 4 °C overnight and mounted on a microscope slide to produce single cells by slightly hitting. Images were captured using a Leica DM5000B microscope with a DFC camera for further analysis in Image J software (Abràmoff *et al.*, 2004).

### Rubisco quantitation

Leaf samples of 1cm were collected from the widest part of a fully expanded leaf 6 after the gas exchange measurements had been taken and then immediately immersed in liquid nitrogen and stored at –80 °C. The leaf area (1cm × leaf width) was calculated and used to determine the quantitation in a unit area. Rubisco extraction was performed by adding 1ml extraction buffer (100mM HEPES, 1mM EDTA, 20mM MgCl_2_, 0.1% (v/v) Triton, 20% (v/v) glycerol, 0.12% (w/w) DTT, pH 7.5) to lysing matrix tubes with additional 1/4″ ceramic spheres (supplied by MP Biomedical). The tubes were put in MPTM FAST PREP®-24 (MP Biomedical) under a maximum vortex speed for 1min to grind the sample. Samples were centrifuged at 13 000×*g* for 10min at 4 °C and the supernatant was then transferred to another Eppendorf tube and stored at –80 °C. Total protein calculation and the SDS-PAGE for separating the large subunit of Rubisco was performed as described by Murchie *et al.* (2002) and pure wheat Rubisco protein was kindly supplied by Martin Parry, Rothamsted Research, UK. The gel was stained in QC Colloidal Coomassie Stain (Bio-Rad) for 1h and destained in water to remove background colour. Gel densitometry was performed using a GS-800 Calibrated Densitometer (Bio-Rad) and Quantity One version 4.4.1 software. The density of Rubisco in 1 µg total protein was calculated from SDS-PAGE densitometry compared with the pure Rubisco reference.

### Statistical analysis

Values were used to perform statistical analysis in GraphPad Prism 6 software. Unpaired *t*-tests were performed to compare two sets of values between mutant plants and their control plants (for instance, the comparison of anatomical structure). Paired *t*-tests were performed for the analysis of matched pairs of values between mutant plants and their control plants (for instance, stomatal conductance under a series of changing APR).

## Results

### Complementation of *hws-1* using *OsEP3*

**Fig. 1.**
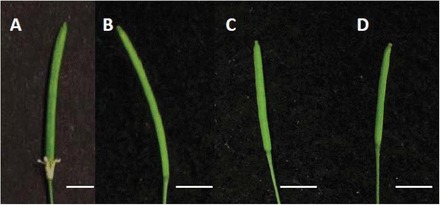
Phenotypic analysis of flowers from *hws-1* and Col-0 plants containing pBI101:*HWS*pro:*EP3.* Green siliques from *hws-1* (A), Col-0 WT (B), *HWS:EP3/hws-1* (C), and *HWS:EP3*/Col-0 WT (D). Sepal fusion in *hws-1*(A) was rescued by expressing *EP3* driven by the *HWS* promoter (C). Scale bar=0.5cm.

To determine whether the *OsEP3* is a functional orthologue of *HWS* the predicted *EP3* exon 2 was cloned using genomic DNA from the Hwasunchalbyeo ecotype and sub-cloned into the vector pBI101.2:*HWS*_pro_ (González-Carranza *et al.*, 2007). A phenotypic comparison of flowers was performed between transgenic plants and their controls. Sepals of *hws-1* flowers were fused into a single whorl and remained attached to siliques throughout maturity and dehiscence (Fig. 1A). Floral organs from *hws-1* plants containing the construct pBI101.2: *HWS*_pro_:*EP3* (Fig. 1C) resembled wild-type plants (Fig. 1B) and exhibited normal floral organ shedding. Similarly, Col-O plants transformed with the pBI101.2: *HWS*_pro_:*EP3* construct were indistinguishable from the wild type (Fig. 1D).

### Photosynthetic assimilation and stomatal conductance in the *ep3* mutant

**Fig. 2.**
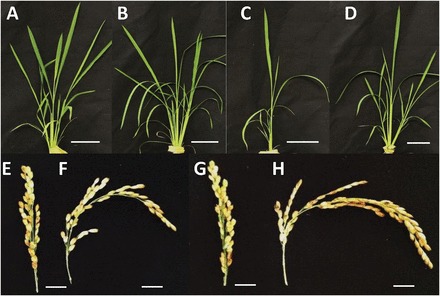
Comparison of aerial tissues (A–D) at the leaf 6 stage and mature panicles (E–H) at the harvest stage from two rice lines carrying a mutation in the *EP3* gene grown in hydroponics. (A, E) *ep3* NMU mutant; (B, F) Hwasunchalbyeo, from which the NMU mutant line was generated; (C, G) *ep3* T-DNA insertion 1C-03432.L; (D, H) Hwayoungbyeo, from which the *ep3* T-DNA insertion 1C-03432.L was generated. Scale bars: (A–D) 10cm; (E–H) 1cm.

Gas-exchange measurements were performed on the fully expanded leaf 6 from the *ep3* NMU mutant, Hwasunchalbyeo, the *ep3* T-DNA insertion 1C-03432.L, and Hwayoungbyeo grown in a hydroponic system (Fig. 2A–D).

**Fig. 3.**
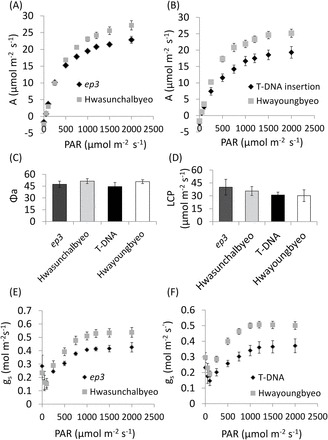
Light-response curve from fully expanded leaf 6 of *ep3* mutants and their WT control plants. (A) A significant decrease of photosynthetic assimilation was found in *ep3* NMU mutant plants compared with Hwasunchalbyeo over 500 µmol m^–2^ s^–1^ PAR, *P* <0.001 (paired *t*-test). (B) Significantly lower photosynthesis was also observed in the *ep3* T-DNA insertion line compared with Hwayoungbyeo over 500 µmol m^–2^ s^–1^ PAR, *P* <0.001 (paired *t*-test). Analysis of apparent quantum yield (C) and light compensation point (D) indicate no significant change. (E, F) Stomatal conductance (*g*_s_) in response to PAR. Error bars in this figure show the SD, *n*=5.

The light-response curves from all four lines showed typical responses but the net photosynthetic assimilation at higher light levels, including the light-saturated rate (*A*_max_) was observed to be lower in the *ep3* NMU mutant and the T-DNA insertion line when compared with Hwasunchalbyeo (unpaired *t*-test, *P* <0.001) and Hwayoungbyeo (unpaired *t*-test, *P* <0.001), respectively (Fig. 3A, B). Apparent quantum yield and light compensation point were calculated from the linear portion of the light-response curve at lower PAR but no significant difference between each gene function disruption line and their WT plants was seen (Fig. 3C). Analysis of the light compensation point also indicated that there was no significant difference between each gene function disruption line and their controls (Fig. 3D). Dark respiration (*R*_d_, µmol m^–2^ s^–1^) was calculated from the light-response curve and there was no significant difference between the *ep3* NMU mutant (1.89±0.40) and Hwasunchalbyeo (2.01±0.26) and between the *ep3* T-DNA insertion (2.18±0.18) and Hwayoungbyeo (1.86±0.34). These results suggest that the lower photosynthetic assimilation observed in the *ep3* NMU mutant and the T-DNA insertion line is neither the consequence of a defect in photosynthetic (quantum) efficiency nor the higher demand of cellular respiration. Stomatal conductance (*g*_s_) calculated from the light-response curve is shown in Fig. 3E, F). *g*_s_ was significantly lower in the *ep3* mutants when compared with their WT plants at a PAR of 500 µmol m^–2^ s^–1^ and above (paired *t*-test, *ep3* NMU mutant versus Hwasunchalbyeo *P* <0.05; paired *t*-test, *ep3* T-DNA insertion versus Hwayoungbyeo *P* <0.001) (Fig. 3E, F).

*A*/*C*_i_ curves were generated to identify the component responsible for the decline in photosynthesis (curves shown in Supplementary Fig. S2 at *JXB* online). Maximum RuBP saturated rate of carboxylation (*V*_cmax_, µmol m^–2^ s^–1^), CO_2_ compensation point (Γ, µmol mol^–1^), maximum rate of electron transport (*J*_max_, µmol m^–2^ s^–1^), and stomatal limitation (*l*) were calculated from the fitted *A*/*C*_i_ curve by the *A*/*C*_i_ Response Curve Fitting 10.0 suite (http://landflux.org/Tools.php) following the Farquhar–von Caemmerer–Berry leaf photosynthesis model (Ethier and Livingston, 2004). There was no significant difference in *J*_max_ between the mutants and their wild-type control plants (Table 1). However, *V*_cmax_ which shows the level of active Rubisco in the leaf and the CO_2_ compensation point (Γ), was significantly lower only in the *ep3* T-DNA insertion when compared with the control (unpaired *t*-test, *P* <0.01 and *P* <0.001, respectively. The proportion of photosynthesis limited by stomatal conductance (*l*) was calculated according to Long and Bernacchi (2003) and was significantly higher in both the *ep3* NMU mutant and the T-DNA insertion line when compared with their control plants (unpaired *t*-test, *ep3* NMU mutant versus Hwasunchalbyeo *P*=0.024; *ep3* T-DNA insertion versus Hwayoungbyeo *P*=0.018). As a lower *g*_s_ was the most consistent effect of *EP3* disruption, this parameter was focused on in more detail.

**Table 1.** Comparison of *V*_cmax_, CO_2_ compensation point, *J*_max_, and stomatal limitationCO_2_ compensation point (Γ, µmol mol^–1^),maximum rate of electron transport (*J*_max_, µmol m^–2^ s^–1^), stomatal limitation (l), and maximum RuBP saturated rate of carboxylation (*V*_cmax_, µmol m^–2^ s^–1^) were calculated from a fitted *A*/*C*_i_ curve by the *A*/*C*_i_ Response Curve Fitting 10.0 suite (available on http://landflux.org/Tools.php). Stomatal limitation (*l*) was calculated using l=(*A*_0_–*A*)/*A*_0_ (*A*_0_ at *C*_i_=400 µmol mol^–1^). An unpaired *t*-test was performed for statistics: * significant at the *P* <0.05 level; ** significant at the *P* <0.01 level; *** significant at the *P* <0.001 level.

	*ep3* NMU mutant	Hwasunchalbyeo	*ep3* T-DNA insertion	Hwayoungbyeo
*V*_cmax_ (µmol m^–2^ s^–1^)	71.18±11.34	81.78±8.68	57.70±7.58**	86.22±11.95
Γ (µmol mol^–1^)	61.98±5.24	60.50±4.36	75.78±5.70***	55.64±3.02
*J*_max_ (µmol m^–2^ s^–1^)	106.5±5.57	112.6±4.32	105.2±8.64	110.4±5.84
Stomatal limitation (*l*) (%)	10.23±5. 42*	4.790±2.94	18.53±2.61*	12.82±5.64

Maximum quantum yield was also measured as dark adapted *F*_v_/*F*_m_ and no differences were seen between the *ep3* NMU mutant and the T-DNA insertion line and their controls, indicating that lowered photosynthesis was not the consequence of a defect in PSII (see Supplementary Fig. S3 at *JXB* online).

To confirm that the *ep3* mutant plants have a lower *g*_s_ and to explore this property in more detail, the gas-exchange measurement was performed using manually altered humidity levels. *g*_s_ was determined under changing humidity conditions while the leaf temperature, CO_2_, and PAR levels were fixed. The *g*_s_ declined in all four plant lines when the humidity decreased (see Supplementary Fig. S4 at *JXB* online). The analysis of *g*_s_ indicated that there were significant decreases in the *ep3* mutant lines when compared with their WT controls (paired t-test, *ep3* NMU mutant versus Hwasunchalbyeo *P*<0.05; *ep3* T-DNA insertion versus Hwayoungbyeo *P*<0.01; see Supplementary Fig. S4 at *JXB* online).The data show consistency in the direction of the response for stomatal conductance in both the humidity and the light response.

### Analysis of stomatal density and stomatal area

To determine any morphological origin of the decline in *g*_s_, stomatal density and stomatal area were determined (see Supplementary Fig. S5 at *JXB* online). Both the adaxial and abaxial surfaces of the middle portion of leaf 6 were investigated. Statistical analysis showed that there was no significant difference in stomatal density between *ep3* functional disruption lines and their WT plants on either the adaxial or abaxial surfaces (Table 2). However, there was a consistent decrease in guard cell area in both the lines compared with the controls. The stomatal guard cell area was significantly smaller in the *ep3* NMU mutant and the T-DNA insertion line on the leaf abaxial surface when compared with Hwasunchalbyeo (unpaired *t*-test, *P* <0.01) and Hwayoungbyeo (unpaired *t*-test, *P* <0.05), respectively (Table 2). On the adaxial surface, a reduction in stomatal guard cell area was only observed in the T-DNA insertion line (unpaired *t*-test, *P* <0.05). The reduced abaxial stomatal guard cells area in the *ep3* NMU mutant is the consequence of a reduction in stomatal width (unpaired *t*-test, *P* <0.001) while a reduced stomatal guard cell length in the T-DNA insertion line (unpaired *t*-test, *P* <0.001) was responsible for the area reduction (Table 2). A decrease in stomatal guard cell length from the adaxial surface was observed in the T-DNA insertion line (unpaired *t*-test, *P* <0.001) (Table 2) and this reduction was responsible for a smaller adaxial guard cell area in the T-DNA insertion line (Table 2). Abaxial- and adaxial-specific responses of stomatal density to alterations in growth irradiance and CO_2_ were observed in a previous study (Hubbart *et al.*, 2013).

**Table 2.** Analysis of stomatal density, stomatal (guard cell) area, length, and widthThe table shows measurements of stomatal distribution and stomatal area, length, and width in the form of ‘mean±SD’ for *n*=40. An unpaired *t*-test was performed for statistics: * significant at the *P* <0.05 level; ** significant at the *P* <0.01 level; *** significant at the *P* <0.001 level.

Lines	Surface	Stomatal number(mm^–2^)	Guard cell length(µm)	Guard cell width(µm)	Guard cell area(µm^2^)
*ep3* NMU mutant	Adaxial	182.5±6.29	20.50±0.41	8.05±0.15	143.53±4.89
Hwasunchalbyeo	Adaxial	190.27±3.93	21.12±0.31	7.67±0.16	137.59±3.46
T-DNA insertion	Adaxial	200.55±5.39	20.72±0.46***	7.82±0.14	143.02±5.34*
Hwayoungbyeo	Adaxial	197.22±5.47	23.35±0.34	8.20±0.17	158.60±5.84
*ep3* NMU mutant	Abaxial	209.72±7.34	19.26±0.41	6.37±0.16***	116.68±3.53**
Hwasunchalbyeo	Abaxial	215.83±7.72	19.26±0.32	7.49±0.13	129.88±3.29
T-DNA insertion	Abaxial	222.22±4.01	19.19±0.31***	7.87±0.14*	133.68±2.94*
Hwayoungbyeo	Abaxial	238.05±8.48	20.24±0.51	7.43±0.12	143.80±3.51

Correlation analysis between stomatal parameters and stomatal conductance was also performed but no significant correlation was observed (see Supplementary Fig. S6 at *JXB* online).

### Leaf anatomy

Alterations in stomatal guard cells area could be indicative of other leaf developmental effects (Hubbart *et al.*, 2013). Therefore, the leaf anatomical structure was analysed using transverse sections taken from the middle area of fully expanded leaf 6 (see Supplementary Fig. S7 at *JXB* online). Statistical analysis showed that there was no significant difference in the following parameters: leaf thickness at minor veins; interveinal distance between the major vein and minor veins; and leaf thickness at the major vein and the width of the major vein (Table 3). Significant changes were observed in the following parameters: the interveinal distance between minor veins was significantly reduced in the *ep3* NMU mutant when compared with Hwasunchalbyeo (unpaired *t*-test, *P* <0.05) (Table 3); the leaf thickness at a bulliform cell position was significantly smaller in the T-DNA insertion line when compared with Hwayoungbyeo (unpaired *t*-test, *P* <0.01) (Table 3); and the width of the minor vein was also significantly reduced in the *ep3* mutant (unpaired *t*-test, *P* <0.01) (Table 3). In all of these changed parameters, the significant changes were only observed in one of the *ep3* functional disruption lines (*ep3* NMU mutant or the T-DNA insertion line).

**Table 3.** Analysis of leaf anatomical structuresThe table shows measurements of leaf anatomical structures from leaf sections and single mesophyll cell preparation in the form of ‘mean±SD’ for *n*=5–40. An unpaired *t*-test was performed for statistics: * significant at the *P* <0.05 level; ** significant at the *P* <0.01 level.

	*ep3* NMU mutant	Hwasunchalbyeo	*ep3* T-DNA line	Hwayoungbyeo
Major vein width (µm)	114.21±5.44	119.05±2.59	115.96±5.88	118.66±3.37
Minor vein width (µm)	46.75±1.39**	41.44±1.13	44.21±1.91	43.19±1.39
Leaf thickness at	174.34±5.44	182.97±2.59	179.80±5.88	176.11±3.37
major vein (µm)				
Leaf thickness at	85.39±1.64	85.33±1.85	87.07±1.80	91.52±1.49
minor vein (µm)				
Leaf thickness at	86.11±2.18	83.07±2.07	80.52±1.69**	88.13±1.64
bulliform cells (µm)				
Interveinal distance between	137.01±4.09	143.16±4.09	122.12±5.08	124.76±2.02
major vein and minor vein (µm)				
Interveinal distance between	139.99±3.03*	150.73±3.19	128.35±4.40	129.57±3.52
minor veins (µm)				
Mesophyll cell area (µm^2^)	279.62±8.35	296.91±8.99	331.40±10.83	347.18±11.73
Mesophyll cell lobe number	7.08±0.28	7.44±0.26	7.36±0.23	7.68±0.243

Measurements of mesophyll cell area and cell shape were performed on single mesophyll cells separated from the fully expanded leaf 6 tissues (see Supplementary Fig. S8 at *JXB* online). The analysis of mesophyll cell area showed that there was no significant difference between the *ep3* functional disruption lines and their WT plants (Table 3). The number of mesophyll cell lobes was also analysed, and no significant difference was observed between the *ep3* functional disruption lines and their WT plants (Table 3).

A significant increase in leaf width on leaf 6 from the *ep3* NMU mutant was observed when compared with Hwasunchalbyeo (unpaired *t*-test, *P* <0.05), however, no significant difference was observed between the T-DNA insertion line and its Hwayoungbyeo control (data not shown).

**Fig. 4.**
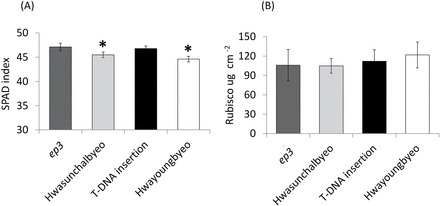
Analysis of SPAD index and Rubisco. Fully expanded leaf 6 from rice plants were used for these measurements. Error bars indicate SD value for *n*=3 in Rubisco and *n*=18 in SPAD. (A) SPAD index of mutant plants and WT control plants, indicating a proxy for chlorophyll content. (B) Analysis of Rubisco concentration per unit leaf area (µg cm^–2^).

Leaf chlorophyll content taken as SPAD values were measured on leaf 6 and statistical analysis indicated that the SPAD value was significantly higher in the *ep3* mutant lines when compared witho their WT plants (unpaired *t*-test, *ep3* NMU mutant versus Hwasunchalbyeo, *P* <0.05; *ep3* T-DNA insertion line versus Hwayoungbyeo, *P* <0.001) (Fig. 4A).

Under optimal conditions, saturating light and ambient CO_2_ concentrations, the content of active Rubisco protein commonly limits a large proportion of photosynthetic rate (Makino, 2011). Our data did not show a consistent lowering of Rubisco activity in both lines, as measured as *V*_cmax_. Hence the Rubisco protein concentration per unit leaf area was measured using a SDS-PAGE gel (see Supplementary Fig. S9 at *JXB* online). There was no significant difference between the *EP3* functional disruption line and their WT plants (Fig. 4B). This result suggests that the reduction in photosynthesis of the *ep3* mutant and the T-DNA insertion line is not caused by differences in the quantity of Rubisco present but may represent a down-regulation of Rubisco activity.

## Discussion

### *OsEP3* is a functional orthologue of the *Arabidopsis HWS* gene

The fused sepal phenotype of the *hws-1* mutant was rescued by expressing the *OsEP3* exon 2 driven by the *HWS* promoter (Fig. 1B). This observation indicates that the exon 2 of *OsEP3* is able to substitute for the function of *HWS* during floral development and confirms that *OsEP3* is a functional orthologue of *At3g61590*. *HWS* has two potential orthologues in rice according to protein sequence similarity, these are *Os01g47050* and *Os02g15950* (*ERECT PANICLE3*). A previous study confirmed that *Os01g47050* is able to complement *hws-1* mutant plants (Z González-Carranza, unpublished data) which indicates that the *HWS* orthologue in rice is duplicated. The phenotypic characteristics of silencing the *Os01g47050* gene are unknown and it would be intriguing to determine the features of a double knockout of both this gene and *Os02g15950* (*ERECT PANICLE3*) to ascertain whether organ fusion and growth are also compromised as observed in *hws-1* (González-Carranza *et al.*, 2007) and in Fig. 2. The protein sequence alignment reported by Piao *et al.* (2009) indicated that there are two exons in *Os02g15950* but only one exon in *HWS* and its orthologues. Interestingly, the second exon of *OsEP3* also starts with an ATG and our study has shown that the second exon of *EP3* is able to rescue the fused sepal phenotype in *hws-1*. Thus, it is possible that the first predicted exon of *EP3* might not be an accurate interpretation of the structure of the gene. However, it is also possible that the *EP3* RNA might undergo alternative splicing. Further analyses need to be carried out to determine which of these alternatives is correct.

### Reduced stomatal guard cell area is associated with a lowered stomatal conductance and photosynthetic capacity in *ep3* mutants

A significantly lower photosynthetic capacity and *g*_s_ was observed in both the *ep3* NMU mutant and the T-DNA insertion line from light-response curves when compared with their WT plants, Hwasunchalbyeo and Hwayoungbyeo, respectively. No consistent change in apparent maximum quantum yield of CO_2_ uptake (Φ_a_), light compensation point (LCP), quantity of Rubisco, *F*_v_/*F*_m_, mesophyll cell size, and lobes were found. However, significant differences were observed in guard cell (stomatal) length and guard cell (stomatal) area (Table 2) and the proportion of photosynthesis limited by stomatal conductance between both the *ep3* functional disruption lines and their WT plants. In this section, the term stomatal area is used to reflect the changes seen in both the length and width of the guard cells (Table 2). Significant differences in some anatomical changes were only observed in the *ep3* NMU mutant or the DNA insertion line (Table 3). The changes only observed in one mutant line may be the consequence of some other mutations in the genome or because the truncated peptides generated in the *ep3* mutant retain some function when only the F-box domain of the protein was translated.

The stomatal densities and area observed here are in line with published values for rice (Ohsumi *et al.*, 2007; Hubbart *et al.*, 2013). It is proposed that the smaller area of guard cells in the mutants with no alteration in stomatal density is, at least partly, responsible for the lowered stomatal conductance which, in turn, caused the lower values of photosynthesis. However, an additional change in stomatal aperture cannot be ruled out. There were no consistent changes in other leaf anatomical changes or in Rubisco protein content, suggesting that the reduction of stomatal area is not the consequence of the general cell size reduction throughout the rice leaves. Stomatal area, density of stomata, and depth of the stomatal pore all have a role in determining conductance values, although the relationships can be complex. For example, stomatal size has been reported to have an inverse relationship with stomatal density in rice (Ohsumi *et al.*, 2007) and across species and genera, including fossil plants (Hetherington and Woodward, 2003). Importantly, stomatal density does not correlate with conductance across a range of rice genotypes but a significant positive correlation between stomatal length and stomatal conductance can be observed (Ohsumi *et al.*, 2007) which support our hypothesis. However, a significant change in length was only observed in the Hwayoungbyeo mutant. An inverse relationship between guard cell size and conductance has been observed in other studies and other species (Lawson and Blatt, 2014; Raven, 2014), but not in this study (see Supplementary Fig. S6 at *JXB* online) or in a survey of rice genotypes (Ohsumi *et al.*, 2007). Rice stomata are small in comparison with other species so it is possible that other factors, including area, density, and depth of the pore are more significant. It is proposed here that the alteration of area with no change in density will reduce the maximum possible pore area per unit leaf area for gas exchange.

To improve photosynthesis and yield under natural environments the speed of stomatal movements is considered to be important especially in fluctuating light (Lawson *et al.*, 2012). Quick responses in terms of stomatal opening and closing were reported to be essential for optimizing water loss and CO_2_ gain (Vico *et al.*, 2011; Lawson *et al.*, 2012). In the *ep3* mutant, smaller stomata may be a benefit in terms of speed of response and reducing the expense of stomatal movement. In the gas exchange measurements the temporal pattern of response (the kinetics of the light-response curve, for example) of the mutant and the wild type was similar in each case: it was only the magnitude of the response that differed, suggesting that there was no substantial change in stomatal movement dynamics over the time-scale used.

### Stomatal conductance, Rubisco, and photosynthesis

A correlation between stomatal conductance and photosynthesis is common (Wong *et al.*, 1979; Farquhar and Sharkey, 1982; Leuning, 1995), however, many previous studies have shown that, under optimal conditions and ambient CO_2_, Rubisco quantity and activation state dominate in the determination of photosynthetic capacity in rice (Makino, 2011; Parry *et al.*, 2013). Rubisco quantity did not show significant differences (Fig. 4B) in this study and it is proposed here that the limitations on CO_2_ diffusion caused by lowered stomatal conductance resulted in a lowered Rubisco activation state in the *ep3* mutant as shown by the *V*_cmax_. This is in accord with previous studies where stomatal conductance was shown to place a limitation on Rubisco activity (Flexas *et al.*, 2006). It is interesting that the *V*_cmax_ was only significantly lower in the T-DNA insertion line (Table 1), indicating that, although stomatal conductance is still the original limiting factor, there may be small differences in the way Rubisco activity is regulated between Hwasunchalbyeo and Hwayoungbyeo.

### F-box protein EP3 and stomatal area

In this study, it is proposed that the reduced stomatal area in *ep3* functional disruption lines leads to a lower photosynthetic assimilation via a reduction in stomatal conductance while no difference was found in stomatal density and the mesophyll cell size (Table 3). Therefore, the *EP3* gene was inferred to play a role in regulating stomatal area. As an F-box protein, EP3 may function as a subunit of ubiquitin E3 ligase to recognize and degrade a certain substrate (Petroski and Deshaies, 2005; Lechner *et al.*, 2006) that is a determinant of stomatal area. Stomatal development has been well studied in *Arabidopsis* and a number of genes have been reported to be involved in the process (Pillitteri and Torii, 2012). Two F-box genes *DROUGHT TOLERANCE REPRESSOR* (*DOR*) (Zhang *et al.*, 2008) and *CONSTITUTIVE PHOTOMORPHOGENIC* (*COP1*) (Kang *et al.*, 2009) were reported to regulate the stomatal ABA response and light-controlled stomatal development, respectively. This also provides evidence that F-box proteins are closely related to stomatal regulatory networks. The pathway controlling stomatal size (*S*) and density (*D*) seems to be linked by observing an inverse relationship between the *S* and *D* under different CO_2_ levels (Franks and Beerling, 2009; Doheny-Adams *et al.*, 2012). The gene *HIGH CARBON DIOXIDE* (*HIC*) was reported to modulate a change in stomatal density in response to elevated CO_2_ (Gray *et al.*, 2000). In this study, a stomatal area decrease has been observed in the *ep3* mutant without disrupting stomatal density, which did not show an inverse relationship. Expression analysis of *EP3* RT-PCR was performed to detect the expression pattern of *EP3.* Expression of *EP3* was detected in Hwayoungbyeo, the *ep3* NMU mutant, and Hwasunchalbyeo leaf tissues (see Supplementary Fig. S10 at *JXB* online). Studies on the *HAWAIIAN SKIRT* (*HWS*) gene in *Arabidopsis* also identified expression in leaf tissue (González-Carranza *et al.*, 2007) and an abnormal distribution of stomata took place in the *hws-1* mutant (Z González-Carranza *et al*., unpublished data), suggesting that the gene may be involved in stomatal lineage. *EP3*, the functional orthologue, might play a similar function as *HWS* in the regulation of stomatal development. In order to understand how EP3 may be involved in stomatal area regulation, it is important to identify the substrate for this F-box protein and our current work on HWS should prove valuable in this regard.

### Summary

*EP3* was confirmed to be a functional orthologue of *HWS* via a complementation test. Observations from gas exchange indicated that a decrease in photosynthesis in the *ep3* mutant was the consequence of a decline in stomatal conductance linked to a reduced guard cell area. *EP3* is likely to play a role in regulating stomatal guard cell area as an F-box protein in the ubiquitin-mediated protein degradation pathway.

## Supplementary data

Supplementary data are available at *JXB* online.

Supplementary Fig. S1. Primers list.

Supplementary Fig. S2. *A*/*C*_i_ curves generated from gas-exchange.

Supplementary Fig. S3. Analysis of dark-adapted *F*_v_/*F*_m_.

Supplementary Fig. S4. Analysis of stomatal conductance (*g*_s_) in response to an alteration in cuvette humidity.

Supplementary Fig. S5. Rice leaf surface impression.

Supplementary Fig. S6. Correlation between stomatal parameters and stomatal conductance.

Supplementary Fig. S7. Rice leaf section showing the measurements of anatomical structure.

Supplementary Fig. S8. Single mesophyll cells from leaves of *ep3* mutants and their wild-type controls.

Supplementary Fig. S9. SDS-PAGE gel running of Rubisco larger subunit and standard sample.

Supplementary Fig. S10. Gene expression analysis of *EP3* in rice tissues.

Supplementary Data

## References

[CIT0001] Abràmoff MD Magalhães PJ Ram SJ 2004 Image processing with Image. J Biophotonics International 11, 36–43.

[CIT0002] Adachi S Nakae T Uchida M Soda K Takai T Oi T Yamamoto T Ookawa T Miyake H Yano M 2013 The mesophyll anatomy enhancing CO_2_ diffusion is a key trait for improving rice photosynthesis. Journal of Experimental Botany 64, 1061–1072. 23349143 10.1093/jxb/ers382

[CIT0003] Bergmann DC Sack FD 2007 Stomatal development. Annual Review of Plant Biology 58, 163–181. 10.1146/annurev.arplant.58.032806.104023 17201685

[CIT0004] Clough SJ Bent AF 1998 Floral dip: a simplified method for *Agrobacterium*-mediated transformation of *Arabidopsis thaliana* . The Plant Journal 16, 735–743. 10069079 10.1046/j.1365-313x.1998.00343.x

[CIT0005] Doheny-Adams T Hunt L Franks PJ Beerling DJ Gray JE 2012 Genetic manipulation of stomatal density influences stomatal size, plant growth and tolerance to restricted water supply across a growth carbon dioxide gradient. Philosophical Transactions of the Royal Society B: Biological Sciences 367, 547–555. 10.1098/rstb.2011.0272 PMC3248714 22232766

[CIT0006] Ethier G Livingston N 2004 On the need to incorporate sensitivity to CO_2_ transfer conductance into the Farquhar–von Caemmerer–Berry leaf photosynthesis model. Plant, Cell and Environment 27, 137–153.

[CIT0007] Farquhar GD Sharkey TD 1982 Stomatal conductance and photosynthesis. Annual Review of Plant Physiology 33, 317–345.

[CIT0008] Feldman AB Murchie EH Leung H Baraoidan M Coe R Yu SM Lo SF Quick WP 2014 Increasing leaf vein density by mutagenesis: laying the foundations for C_4_ rice. PLoS One 9, e94947. 24760084 10.1371/journal.pone.0094947 PMC3997395

[CIT0009] Flexas J Barbour MM Brendel O Cabrera HM Carriquí M Díaz-Espejo A Douthe C Dreyer E Ferrio JP Gago J 2012 Mesophyll diffusion conductance to CO_2_: an unappreciated central player in photosynthesis. Plant Science 193, 70–84. 22794920 10.1016/j.plantsci.2012.05.009

[CIT0010] Flexas J Ribas Carbó M Bota J Galmés J Henkle M Martínez Cañellas S Medrano H 2006 Decreased Rubisco activity during water stress is not induced by decreased relative water content but related to conditions of low stomatal conductance and chloroplast CO_2_ concentration. New Phytologist 172, 73–82. 16945090 10.1111/j.1469-8137.2006.01794.x

[CIT0011] Franks P Beerling D 2009 CO_2_-forced evolution of plant gas exchange capacity and water-use efficiency over the Phanerozoic. Geobiology 7, 227–236. 19338614 10.1111/j.1472-4669.2009.00193.x

[CIT0012] González-Carranza ZH Rompa U Peters JL Bhatt AM Wagstaff C Stead AD Roberts JA 2007 *HAWAIIAN SKIRT*: an F-box gene that regulates organ fusion and growth in *Arabidopsis* . Plant Physiology 144, 1370–1382. 17496113 10.1104/pp.106.092288 PMC1914148

[CIT0013] Gray JE Holroyd GH van der Lee FM Bahrami AR Sijmons PC Woodward FI Schuch W Hetherington AM 2000 The HIC signalling pathway links CO_2_ perception to stomatal development. Nature 408, 713–716. 11130071 10.1038/35047071

[CIT0014] Hetherington AM Woodward FI 2003 The role of stomata in sensing and driving environmental change. Nature 424, 901–908. 12931178 10.1038/nature01843

[CIT0015] Hubbart S Bird S Lake JA Murchie EH 2013 Does growth under elevated CO_2_ moderate photoacclimation in rice? Physiologia Plantarum 148, 297–306. 23020599 10.1111/j.1399-3054.2012.01702.x

[CIT0016] Kang C-Y Lian H-L Wang FF Huang JR Yang HQ 2009 Cryptochromes, phytochromes, and COP1 regulate light-controlled stomatal development in Arabidopsis. The Plant Cell 21, 2624–2641. 19794114 10.1105/tpc.109.069765 PMC2768914

[CIT0017] Kim TH Böhmer M Hu H Nishimura N Schroeder JI 2010 Guard cell signal transduction network: advances in understanding abscisic acid, CO_2_, and Ca^2+^ signaling. Annual Review of Plant Biology 61, 561–591. 10.1146/annurev-arplant-042809-112226 PMC3056615 20192751

[CIT0018] Kusumi K Hirotsuka S Kumamaru T Iba K 2012 Increased leaf photosynthesis caused by elevated stomatal conductance in a rice mutant deficient in SLAC1, a guard cell anion channel protein. Journal of Experimental Botany 63, 5635–5644. 22915747 10.1093/jxb/ers216 PMC3444276

[CIT0019] Lawson T Blatt MR 2014 Stomatal size, speed, and responsiveness impact on photosynthesis and water use efficiency. Plant Physiology 164, 1556–1570. 24578506 10.1104/pp.114.237107 PMC3982722

[CIT0020] Lawson T Kramer DM Raines CA 2012 Improving yield by exploiting mechanisms underlying natural variation of photosynthesis. Current Opinion in Biotechnology 23, 215–220. 22296828 10.1016/j.copbio.2011.12.012

[CIT0021] Lechner E Achard P Vansiri A Potuschak T Genschik P 2006 F-box proteins everywhere. Current Opinion in Plant Biology 9, 631–638. 17005440 10.1016/j.pbi.2006.09.003

[CIT0022] Leuning R 1995 A critical appraisal of a combined stomatal-photosynthesis model for C_3_ plants. Plant, Cell and Environment 18, 339–355.

[CIT0023] Long S Bernacchi C 2003 Gas exchange measurements, what can they tell us about the underlying limitations to photosynthesis? Procedures and sources of error. Journal of Experimental Botany 54, 2393–2401. 14512377 10.1093/jxb/erg262

[CIT0024] MacAlister CA Ohashi-Ito K Bergmann DC 2006 Transcription factor control of asymmetric cell divisions that establish the stomatal lineage. Nature 445, 537–540. 17183265 10.1038/nature05491

[CIT0025] Makino A 2011 Photosynthesis, grain yield, and nitrogen utilization in rice and wheat. Plant Physiology 155, 125–129. 20959423 10.1104/pp.110.165076 PMC3014227

[CIT0026] Markwell J Osterman JC Mitchell JL 1995 Calibration of the Minolta SPAD-502 leaf chlorophyll meter. Photosynthesis Research 46, 467–472. 24301641 10.1007/BF00032301

[CIT0027] McKown AD Dengler NG 2007 Key innovations in the evolution of Kranz anatomy and C_4_ vein pattern in *Flaveria* (Asteraceae). American Journal of Botany 94, 382–399. 21636408 10.3732/ajb.94.3.382

[CIT0028] Murchie E Hubbart S Peng S Horton P 2005 Acclimation of photosynthesis to high irradiance in rice: gene expression and interactions with leaf development. Journal of Experimental Botany 56, 449–460. 15647315 10.1093/jxb/eri100

[CIT0029] Murchie EH Yang J Hubbart S Horton P Peng S 2002 Are there associations between grain filling rate and photosynthesis in the flag leaves of field grown rice? Journal of Experimental Botany 53, 2217–2224. 12379789 10.1093/jxb/erf064

[CIT0030] Ohashi-Ito K Bergmann DC 2006 Arabidopsis FAMA controls the final proliferation/differentiation switch during stomatal development. The Plant Cell 18, 2493–2505. 17088607 10.1105/tpc.106.046136 PMC1626605

[CIT0031] Ohsumi A Kanemura T Homma K Horie T Shiraiwa T 2007 Genotypic variation of stomatal conductance in relation to stomatal density and length in rice (*Oryza sativa* L.). Plant Production Science 10, 322–328.

[CIT0032] Parry MA Andralojc PJ Scales JC Salvucci ME Carmo-Silva AE Alonso H Whitney SM 2013 Rubisco activity and regulation as targets for crop improvement. Journal of Experimental Botany 64, 717–730. 23162118 10.1093/jxb/ers336

[CIT0033] Petroski MD Deshaies RJ 2005 Function and regulation of cullin-RING ubiquitin ligases. Nature Reviews Molecular Cell Biology 6, 9–20. 10.1038/nrm1547 15688063

[CIT0034] Piao R Jiang W Ham TH Choi MS Qiao Y Chu SH Park JH Woo MO Jin Z An G 2009 Map-based cloning of the *ERECT PANICLE 3* gene in rice. Theoretical and Applied Genetics 119, 1497–1506. 19756471 10.1007/s00122-009-1151-x

[CIT0035] Pillitteri LJ Sloan DB Bogenschutz NL Torii KU 2006 Termination of asymmetric cell division and differentiation of stomata. Nature 445, 501–505. 17183267 10.1038/nature05467

[CIT0036] Pillitteri LJ Torii KU 2012 Mechanisms of stomatal development. Annual Review of Plant Biology 63, 591–614. 10.1146/annurev-arplant-042811-105451 22404473

[CIT0037] Pyke K Leech R 1987 The control of chloroplast number in wheat mesophyll cells. Planta 170, 416–420. 24232973 10.1007/BF00395035

[CIT0038] Raven JA 2002 Selection pressures on stomatal evolution. New Phytologist 153, 371–386. 10.1046/j.0028-646X.2001.00334.x 33863217

[CIT0039] Raven JA 2014 Speedy small stomata? Journal of Experimental Botany 65, 1415–1424. 24609500 10.1093/jxb/eru032

[CIT0040] Sharma R De Vleesschauwer D Sharma MK Ronald PC 2013 recent advances in dissecting stress-regulatory crosstalk in rice. Molecular Plant 6, 250–260. 23292878 10.1093/mp/sss147

[CIT0041] Smillie I Pyke K Murchie E 2012 Variation in vein density and mesophyll cell architecture in a rice deletion mutant population. Journal of Experimental Botany 63, 4563–4570. 22685308 10.1093/jxb/ers142 PMC3421994

[CIT0042] Vico G Manzoni S Palmroth S Katul G 2011 Effects of stomatal delays on the economics of leaf gas exchange under intermittent light regimes. New Phytologist 192, 640–652. 21851359 10.1111/j.1469-8137.2011.03847.x

[CIT0043] Wong S Cowan I Farquhar G 1979 Stomatal conductance correlates with photosynthetic capacity. Nature 282, 424–426.

[CIT0044] Xing Y Zhang Q 2010 Genetic and molecular bases of rice yield. Annual Review of Plant Biology 61, 421–442. 10.1146/annurev-arplant-042809-112209 20192739

[CIT0045] Zhang Q 2007 Strategies for developing green super rice. Proceedings of the National Academy of Sciences, USA 104, 16402–16409. 10.1073/pnas.0708013104 PMC2034246 17923667

[CIT0046] Zhang Ye Xu W Li Z Deng XW Wu W Xue Y 2008 F-box protein DOR functions as a novel inhibitory factor for abscisic acid-induced stomatal closure under drought stress in *Arabidopsis* . Plant Physiology 148, 2121–2133. 18835996 10.1104/pp.108.126912 PMC2593669

[CIT0047] Zhu XG Long SP Ort DR 2010 Improving photosynthetic efficiency for greater yield. Annual Review of Plant Biology 61, 235–261. 10.1146/annurev-arplant-042809-112206 20192734

