# Supplementary material for: Decreased photosynthesis in the erect panicle 3 (ep3) mutant of rice is associated with reduced stomatal conductance and attenuated guard cell development
Source: J Exp Bot. 2015 Jan 11;66(5):1543–52. doi: 10.1093/jxb/eru525 (PMC4339609; doi:10.1093/jxb/eru525)
Supplement: Supplementary Data [file supp_66_5_1543__index.html]

Decreased photosynthesis in the erect panicle 3 (ep3) mutant of rice is associated with reduced stomatal conductance and attenuated guard cell development — Decreased photosynthesis in the erect panicle 3 (ep3) mutant of rice is associated with reduced stomatal conductance and attenuated guard cell development — Supplementary Data 

# Decreased photosynthesis in the *erect panicle 3* (*ep3*) mutant of rice is associated with reduced stomatal conductance and attenuated guard cell development

## Supplementary Data

Data files

**Files in this Data Supplement:**

- Supplementary Data - Supplementary Data
