# Supplementary material for: Decreased photosynthesis in the erect panicle 3 (ep3) mutant of rice is associated with reduced stomatal conductance and attenuated guard cell development
Source: J Exp Bot. 2015 Jan 11;66(5):1543–52. doi: 10.1093/jxb/eru525 (PMC4339609; doi:10.1093/jxb/eru525)
Supplement: Supplementary Data [file supp_eru525_jexbot135053_file001.pdf]

Decreased photosynthesis in the *erect panicle 3* (*ep3*) mutant of rice is associated with reduced stomatal conductance and attenuated guard cell development

Hongyang Yu, Erik H Murchie, Zinnia H Gonzalez-Carranza, Kevin A Pyke, Jeremy A Roberts*

Primer name	Sequences 5' to 3'	Intended purpose
FCAPsEP3	TCGTGTGCAACCCAATAACTAAGGTCT	Primers used to test ep3 NMU mutant
RCAPsEP3	TAGTTCAGTGAAGGGAGACACCCATGTA	
F2EP3	CATGGGTGTCTCCCTTCACT	Primers used to test the ep3 T-DNA insertion line 1C-03432.
R2EP3	GGTGAATGGCATCGTTTGAA	
FLB	CTAGGAAGCAATGTCCAGCC	
ForEP3	ATGGGGTCAGAGGAGTGGGAG	Primers used to construct pBI101.2:HWSpro:EP3 and sequencing
RevEP3	CTAGGAAGCAATGTCCAGCC	
ForEP3BamHI	CGGGATCCATGGGGTCAGAGGAGTGGGAG	
RevEP3SmaI	TCCCCCGGGCTAGGAAGCAATGTCCAGCC	
ForHWS5UTR	AACCCGCATTTCCTCTCGC	
RevGUS90	GCTTTCCCACCAACGCTG	
SSLPHSfor	GAGAGAGGCTTGTGATTGTCGGAG	*HWS* specific primers used to identify *hws-1* from WT
SSLPHSrev	GTGCCACTACTCGCGAAAACCTCG	

Figure S1. The list of primers.

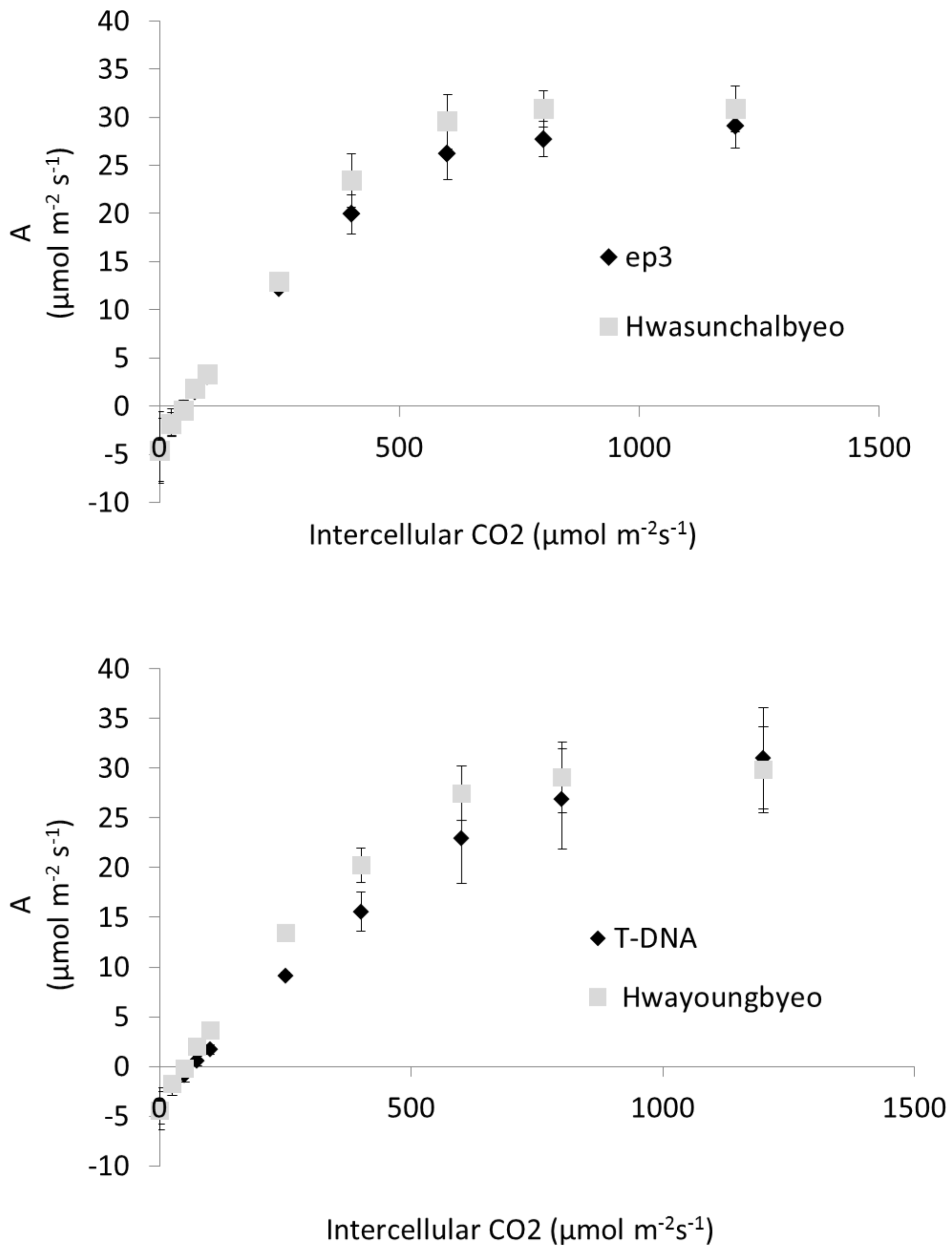

Figure S2. A/Ci curves generated from gas-exchange and the comparison between *ep3* NMU mutant and Hwasunchalbyeo; between the T-DNA insertion line and Hwayoungbyeo.

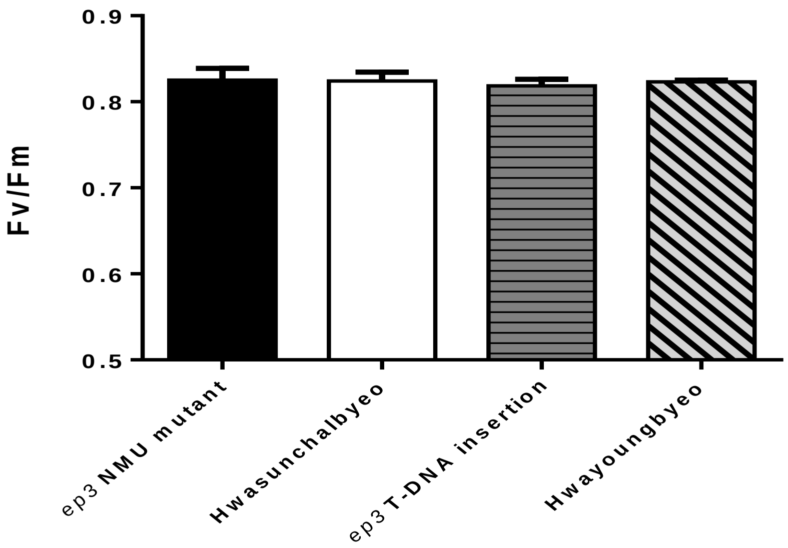

Figure S3. Analysis of dark adapted Fv/Fm from *ep3* NMU mutant, Hwasunchalbyeo, *ep3* T-DNA insertion 1C-03432.L and Hwayoungbyeo.

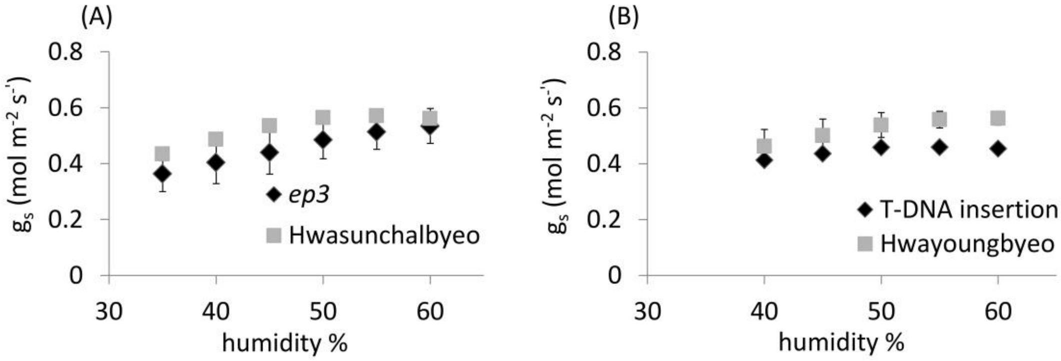

Figure S4. Analysis of stomatal conductance (g_s) in response to an alteration in cuvette humidity. A significant decrease in g_s was observed in both *ep3* NMU mutant plants (A) (P<=0.001, paired t-test) and T-DNA insertion plants (B) (P<0.01, paired t-test) when compared with Hwasunchalbyeo and Hwayoungbyeo respectively. Error bars in this figure show the SD, n=5.

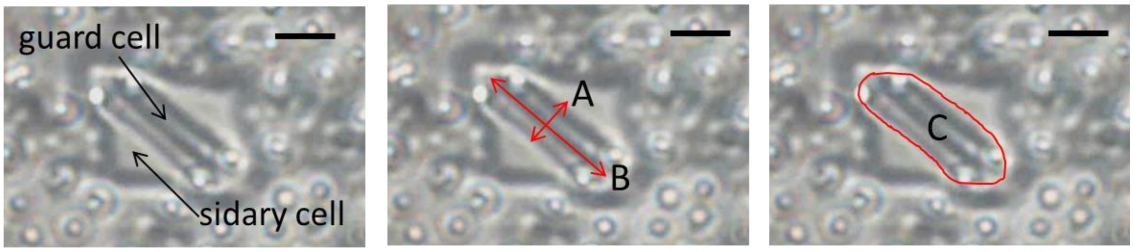

Figure S5. Rice leaf surface impression showing the measurements of stomatal length (A), width (B) and area (C). Scale bar = 10 µm.

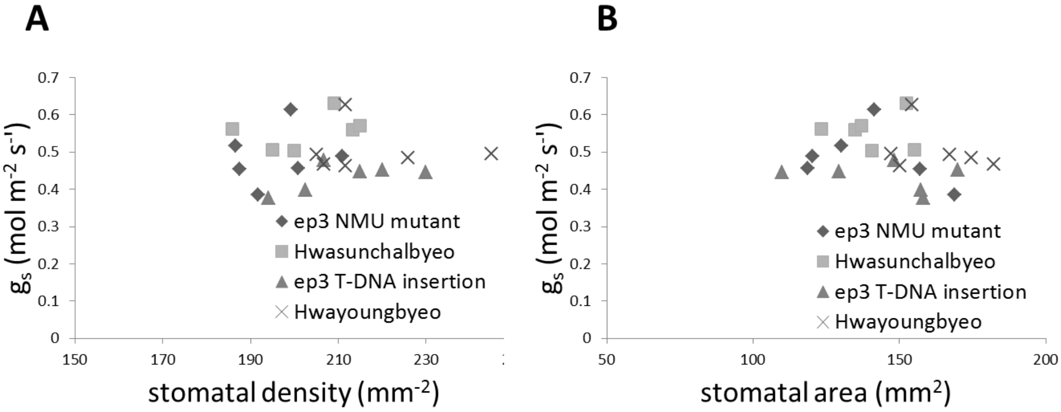

Figure S6. (A) Correlation between stomatal density and stomatal conductance at PAR 1000 µmol m^{-2} s^{-1} (10% blue), relative humidity 50%. Each point represents one biological replicate for g_s and mean of 6 replicates for stomatal density. Stomatal density was calculated from both side of widest part of leaf-6 surface. Regression analysis didn't show significant correlation at 95% level using equation y=0.0003x+0.4432, R^2=0.0026. (B)Correlation between stomatal area and stomatal conductance at PAR 1000 µmol m^{-2} s^{-1} (10% blue), relative humidity 50%. Each point represents one biological replicate for g_s and mean of 12 individuals for stomatal area. Stomatal area was calculated from both side of widest part of leaf-6 surface. Regression analysis didn't show significant correlation at 95% level using equation y=-0.0007x+0.6029, R^2=0.0396.

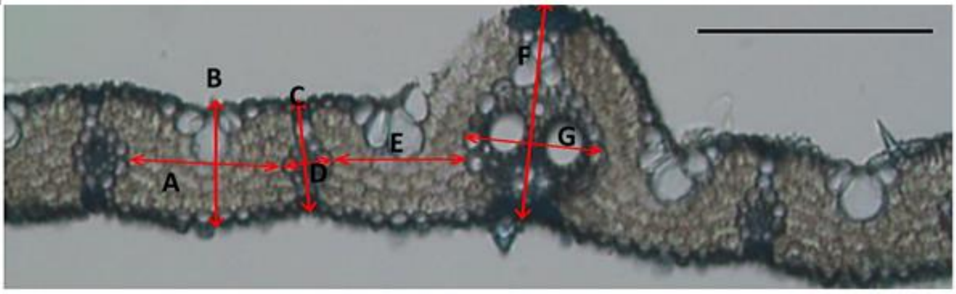

Figure S7. Rice leaf section showing the measurements of anatomical structure (A)The interveinal distance between minor veins; (B) leaf thickness at bulliform cells; (C) leaf thickness at minor vein; (D) width of minor vein; (E) interveinal distance between major vein and minor vein; (F) leaf thickness at major vein; (G) width of major vein. Scale bar = 200 µm.

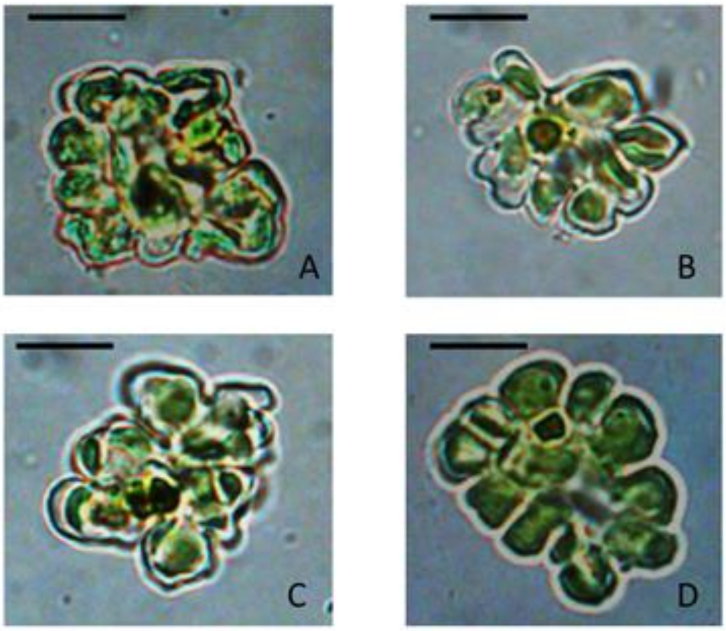

Figure S8. Single mesophyll cells preparation from (A) *ep3* NMU mutant; (B) Hwasunchalbyeo; (C) *ep3* T-DNA insertion 1C-03432.L; (D) Hwayoungbyeo. The scale bars indicate 10 µm.

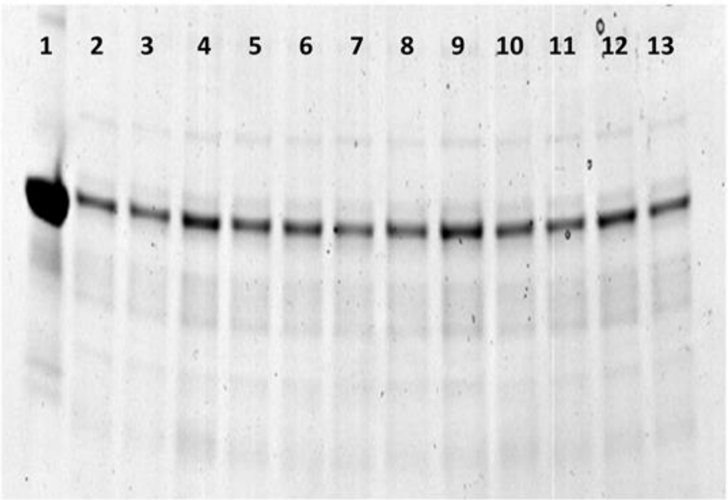

Figure S9. SDS-PAGE gel running of Rubisco larger subunit and standard sample. Lane 1, the standard Rubisco; lane 2-4, the three replicates of ep3 NMU mutant; 5-7, the three replicates of Hwasunchalbyeo; 8-10, the three replicates ofep3 T-DNA insertion 1C-03432.L; 11-13,Hwayoungbyeo.

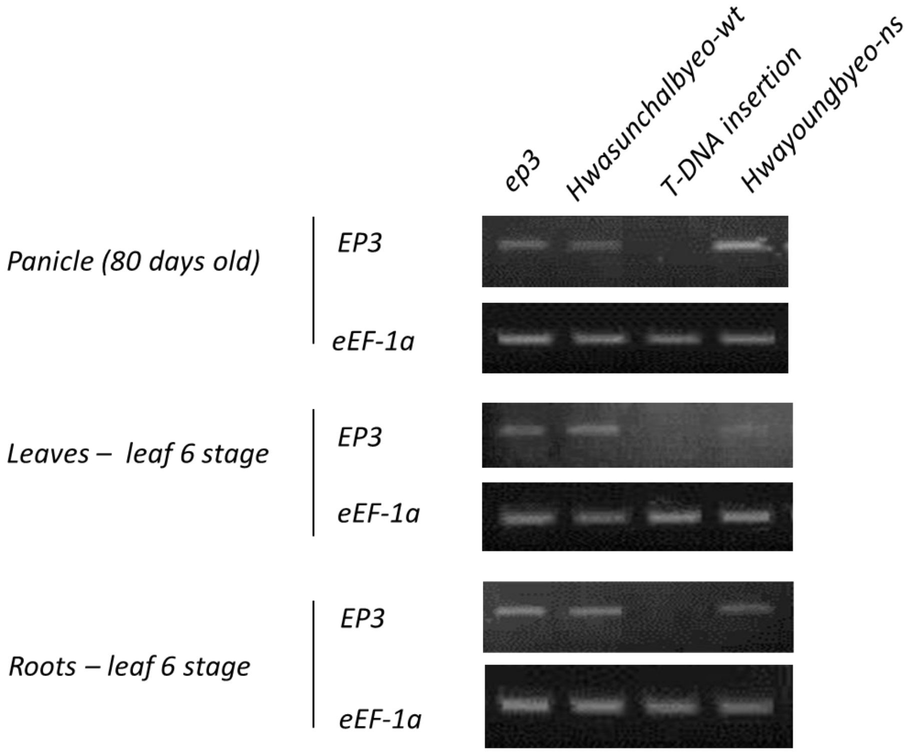

Figure S10. Gene expression analysis of *EP3* in different rice tissues. cDNA templates were generated by reverse-transcription of RNA extracted from rice tissues, including panicles from 80 days old plants, leaves from leaf-6-stage plants and root tissues from leaf-6-stage plants. Rice *eEF-1α* was used as the control gene. In the T-DNA insertion line, no transcripts of *EP3* were observed as the primer was designed to cross the T-DNA insertion.
